# Indigenous Storytelling and Participatory Action Research: Allies Toward Decolonization? Reflections From the Peoples’ International Health Tribunal

**DOI:** 10.1177/2333393615580764

**Published:** 2015-04-20

**Authors:** C. Susana Caxaj

**Affiliations:** 1 UBC Okanagan, Kelowna, British Columbia, Canada

**Keywords:** America, Central, America, South, health promotion, narrative inquiry, participatory action research, power / empowerment, research, collaborative, stories / storytelling, community capacity and development, politics

## Abstract

Storytelling, in its various forms, has often been described as a practice with great emancipatory potential. In turn, Indigenous knowledge shows great promise in guiding a participatory action research (PAR) methodology. Yet these two approaches are rarely discussed in relation to one another, nor, has much been written in terms of how these two approaches may work synergistically toward a decolonizing research approach. In this article, I report on a community-driven knowledge translation activity, the Peoples’ International Health Tribunal, as an exemplar of how narrative and PAR approaches, guided by local Indigenous knowledge, have great potential to build methodologically and ethically robust research processes. Implications for building globally relevant research alliances and scholarship are further discussed, particularly in relation to working with Indigenous communities.

## Introduction

Storytelling, in its various forms, has long been championed as a rich tool for justice-seeking, truth-telling, and Indigenous self-determination (Brown & Strega, 2005; Corntassel, 2009; Smith, 1999). In the global North, research institutes and organizations have pointed to principles of participatory action research (PAR) as key strategies for carrying out research with Indigenous populations in a respectful manner (Canadian Institute of Health Research, 2009; Estey, Smylie, & Macauley, 2009). Furthermore, Indigenous scholars have proposed that principles of PAR reflect an important starting point toward tackling important issues of representation, power, and community benefit (Blodgett, Schinke, Smith, Peltier, & Pheasant, 2011). There is very little documentation, however, of how these two approaches can work in synchrony to develop collaborative research initiatives.

The most familiar storied approaches to research in the academic world are based in a Western school of thought that may be at odds with, negate or minimize local Indigenous epistemologies and ontologies (Brunanski, 2009; Caxaj & Berman, 2014; Stewart, 2009). Yet Indigenous scholars and others working with Indigenous communities are carrying out storied approaches to research, sometimes in contrast to more traditional constructs of narrative research, a continuation or reclamation of unique Indigenous histories (Kovach, 2009; LaDuke, 2005; McLeod, 2000), and other times, by refining or modifying narrative inquiry or analysis to honor Indigenous ways of knowing (Brunanski, 2009; Stewart, 2009; Williams, Labonte, & O’Brien, 2003). Carrying out research with Indigenous communities that incorporates Indigenous storied methodologies can help develop rich, locally relevant insights that may better guide culturally responsive understandings of health experiences.

Furthermore, Indigenous teachings and epistemologies can uniquely guide research activities in such a way that can complement or enrich a PAR methodology. Indigenous values and principles can shed light on ethical and methodological considerations outside of the purview of standard academic institutes (Smith, 1999). Furthermore, they can provide important guidance to ensure high ethical conduct and a more rigorous research design.

In this article, I will describe a knowledge translation initiative that emerged from my doctoral work—an anti-colonial narrative project informed by PAR principles. Shaped by a critical paradigm and local Indigenous knowledge, this research was intended to build a sense of reciprocity, local community benefit, and ultimately, to affect change. Here, I will reflect on my efforts to work with community leaders to incorporate local Indigenous knowledge and participation into research deliverables through an integrated knowledge translation process. In doing so, I seek to champion a synchronized anti-colonial narrative (and) participatory research approach as a rigorous and ethically robust possibility for carrying out research with Indigenous populations.

## Western and Indigenous Storytelling: Divergent Paths, Converging Possibilities

Narrative as a field of study in North America emerged post World War II as a result of various social factors. Among them, the narrative turn in the human sciences, growing identity movements (e.g., race, ethnicity, gender, sexuality, ability), the rising popularity of self-help and talk-show mediums as well as self-performance through electronic and varied art forms (Langellier, 2011). Perhaps as a result of these factors, narrative within the mainstream Western academy is typically understood and defined as a bounded and structured tool or practice with particular components, mechanisms, and outputs in which, storytelling, the performance of narrative, is primarily an expression or process of *self—*individual discovery and meaning-making (Zipes, 2013). For instance, Cavarero (2014), describes a “narratable self” that carries within a “life story [that is] unique and belongs to him or her alone” (p. xvi). Lambert (2013) emphasizes the assertion of personal agency and authority in storytelling, and Zipes discusses “grammatical rules,” strategies, techniques, and insights required to develop plots that assert *one’s* goals. Of particular influence, the construction of narrative as a series of actions within a temporal framework, such as Labov and Waletzky’s (1967) abstract, orientation, evaluation, complication, resolution, and coda, continues to shape the field of narrative study and narrative research.

As noted by Indigenous Scholars, these notions and frameworks reveal basic aspects of a Western school of thought—that is, assumptions about the world and the nature of knowledge that originate in settler/colonial practices. These assumptions may present as incommensurable truths from several Indigenous standpoints. For instance, a focus on human agency on the world may construct a dichotomy of human and nature that is contrary to Indigenous knowledges that champion the interconnectedness and the relational aspect of the universe. Instead, humans are positioned as detached observers from their natural environment or land—an assumption that may be at odds with Indigenous peoples who see themselves in a sacred and intimate relationship with their local environments (Grande, 2008; Kovach, 2009). Furthermore, a focus on plot and temporality may be incompatible with Indigenous lenses that view reality as both cyclical and rooted to local spaces and environments (Castellano, 2008; Caxaj & Berman, 2014).

Further contributing to these ontological disparities in narrative practices, are popular postmodern lenses that some Indigenous scholars see as obfuscating or neglecting the material realities of diverse Indigenous peoples throughout the world. For instance, a focus on identity, representation, and deconstruction divorced from materiality among some critical theorists, may detract from the most pressing issues for Indigenous peoples, which may include access and control of tribal land, poverty, and the destruction of the environment (Grande, 2008). Thus, many postmodern approaches, as currently practiced, may hold little relevance to many Indigenous communities as they reflect the values of relatively privileged populations, in Grande’s words a “theory of property holders” (p. 148). And more generally, even critical theories that account for material realities and contexts reflect a different ontological viewpoint that does not neatly fit with diverse Indigenous realities of interconnectedness, historical legacy, and spirituality (Caxaj, Berman, Varcoe, Ray & Restoule, 2012); Grande, 2008; McIsaac, 2008).

In contrast, Indigenous storytelling is grounded in a unique history and trajectory, revealing value-systems and ways of knowing of diverse Indigenous peoples. Indigenous storytelling, while unique to particular homelands and ancestors of specific peoples, has commonly been described as “lived values that form the basis for Indigenous governance and regeneration” (Corntassel, 2009, p. 2); practices of nurturing and teaching; acts of resistance that can communicate colonial injustices such as residential schools, dispossession, and genocide (Thomas, 2005); and acts that enable multiple meanings and truths to be heard, and, have the power to define what and how knowledge is created (Bishop, 1999). Indigenous notions of storytelling reflect common contours of Indigenous ontologies and epistemologies, such as an assumption of the embodied and timeless nature of knowledge (McIsaac, 2008); knowing through multiplicity, holism, and experience (Castellano, 2008); and a cosmic belonging and responsibility to the earth (Grande, 2008; LaDuke, 2005).

A particular storytelling approach originating among Mestiz@/Indigenous actors in Latin America facing colonial and political violence is that of a *testimonio.* Testimonios, described as *narraciones de* urgencia (emergency or urgent narratives) are a means to bear witness to injustices through spoken or written word. These narratives, even when using the use of “I,” are not removed from the contexts and peoples that jointly experience/are complicit in these stories. That is, testimonios embody a subaltern space that claims authority through virtue of marginality and lived experience, which ultimately, enables the erasure of “the author” (as an attained cultural status) that is so prevalent in Western narrative (Beverley, 2008). In these ways, one can see overlapping values of interconnectedness, justice-seeking, truth-telling, resistance, and survival in both testimonios and other Indigenous storytelling practices.

Given the divergent worldviews that inform narrative and storytelling, researchers working within a Western institution must address ontological tensions with transparency and respect to develop a study that can be of relevance to Indigenous partners. Different notions of what storytelling entails, and how it will frame peoples’ experiences and the ultimate aims of the research, must be developed collaboratively. One strategy is to draw from Western narrative researchers who articulate storied principles that are complementary to Indigenous worldviews. For instance, Clandinin and Connelly (1990) have highlighted the process of narrative inquiry as *doing*; a lived context and a continuous outcome resulting from collaborative intimacy that involves the negotiation of a shared narrative unity. A more crucial component of carrying out meaningful research with Indigenous communities however, involves following protocols and practices for mutuality, power-sharing, and reciprocity. PAR has been championed as an approach that can center local knowledge, involving Indigenous partners to be full collaborators in the research process. PAR involves partnering with communities/participants to carry out research activities and develop research goals. Foundational to this methodology, is an overt need to address power dynamics through trust, relationship-building, and long-term commitment to community benefit (Blodgett et al., 2011). Through this approach, the process of knowledge creation can shift away from the mainstream, and instead, articulate a knowing that reflects Indigenous lives and values (Bartlett, Iwasaki, Gottlieb, Hall, & Mannell, 2007).

## Study Background

From August to November of 2009, I spent 4 months in the community of San Miguel Ixtahuacán, a municipality in the Western highlands of Guatemala, carrying out research. Through this research, I aimed to examine (a) the impact of mining operations on local community health (i.e., relational, collective health) and (b) the role of resistance in promoting community health in the region. In consultation with community leaders, I focused on the experiences of 14 villages in the municipality and was able to recruit 15 men and 41 women from these different locations. Participants were presented with different choices for carrying out data collection. All chose to participate in group conversations with 4 individuals also choosing to participate in one-on-one interviews in addition to their focus group participation. Consent was sought at multiple levels—first, by presenting a proposal to community leaders, then, obtaining consent at each village of interest, and lastly, at the individual level. Individual participants were often nominated during initial group meetings by the collective, and in other cases, individuals simply volunteered themselves during these gatherings. Individual consent was carried out during each subsequent meeting with participants, as well as at particular times during the course of an interview.

Through three to five visits in each participating village, I was able to develop rich and in-depth storied data, and further, to engage in a collaborative data analysis process. This process involved beginning each subsequent village meeting by presenting a thematic summary of the issues that had been discussed during previous visits. Then, I would ask questions of participants to encourage participants to refute, refine, or enrich these preliminary impressions. For instance, in one village, I provided a summary of previously shared experiences of poverty, food insecurity, and concern for the future due to the impact of local mining operations. In response, one woman stated that I had neglected to include the connection between poverty, seasonal work, and the mining company’s rhetoric of development. She saw this rhetoric as a direct assault on the suffering of her fellow community members and felt that it needed to be highlighted in the analysis. In this way, participants co-constructed the emerging findings, adding emphasis, refining and restorying findings in such a way that felt true to their lived realities. In addition, participants were enthusiastic about developing strategies to mobilize these typically collectively shared stories, to affect change in their communities.

These village-level meetings culminated in a community-wide forum where emerging findings were again shared with local participants, friends, and neighbors. Following the presentation of these preliminary findings, again, feedback was sought from community members. Community members were also given paper copies of these emerging findings so that they could provide feedback to the researcher at a later date. Then, in the spirit of previous village-level meetings, the meeting changed in focus to consider potential strategies for action. Several proposals were suggested, but ultimately consensus was reached to carry out the Peoples’ International Health Tribunal. This event would be a social (i.e., popular) moral forum, where the company operating in Guatemala (Vancouver-based Goldcorp), would be “tried.”

Along with these community-based activities, academic publications also detailed some of the health impacts identified by community members describing: (a) complex oppressive forces shaping the systemic influence of mining operations on the community at large (Caxaj, Berman, Restoule, Varcoe & Ray, 2013), (b) collective experiences of *climate of fear and discord* (i.e., mistrust, loss of community harmony, conflict) and embodied expressions of distress (i.e., multi-dimensional—spiritual, physical, psychological—suffering) resulting from the presence of mining operations (Caxaj, Berman, Varcoe, Ray, Restoule, 2014), and (c) multiple modes and sources of community resistance, strategies, and strengths used by individuals and groups to promote well-being amid these challenges (Caxaj, Berman, Ray, Restoule, Varcoe, 2014). Overall, mining operations in the region manifested as an acute mechanism of social unraveling in the community, threatening the relational well-being of the community at large, as well as the holistic health of individual residents (Caxaj et. al, 2014a; Caxaj et. al, 2014b)).

**Figure 1.**
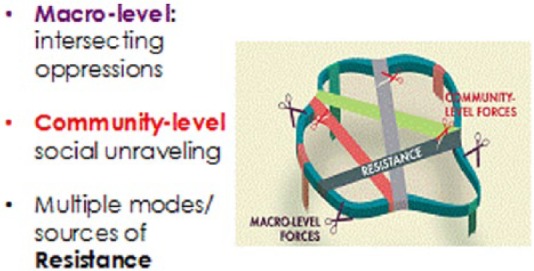
Vulnerabilities and resistance in light of health threats due to mining operations.

This social unraveling or undoing of the social fabric of the community was also reflected in the paradox of community resistance. Local residents reported engaging in various acts of resistance that were fundamental for the health and well-being of their community. However, outspoken individuals possessing the qualities to demonstrate leadership through resistance were often the most vulnerable to targeted persecution endangering their safety and well-being. Furthermore, shared spiritual and cultural ontologies, epistemologies, and identities provided sources of strength and motivation in building healthy spaces and health promoting capacities among community members. Yet through their influence on cultural community structures and institutions, land-based economies, spiritual practices, and the relationship to the land, these same community elements were under threat by the presence of local mining operations. The complex relationship between macro-level findings (purple scissors), community-level findings (red scissors), and community strengths/resistance (inner ribbons) are illustrated in Figure 1. This diagram illustrates the inseparable and layered contexts in which community members experienced health threats occasioned and exacerbated by local mining operations. The community has unique strengths and capacities that can promote well-being and serve as protective elements to various systemic and community-level threats. But, ultimately, oppressive intersecting contexts/histories and immediate experiences of violence, uncertainty, and distress present an overwhelming challenge to the collective well-being and social fabric of the community. Resistance is never obsolete, but without addressing the larger systemic factors, in many ways reflected in the presence/practices of local mining operations, the communities’ well-being will continue to be under significant threat (Caxaj et. al, 2013; Caxaj et. al, 2014b). These findings point to the need to be aware of the multi-faceted nature of threats as well as the strengths and resistance of mining-affected communities to adequately respond to their health needs/priorities.

## The Peoples’ International Health Tribunal

Inspired by the Water Tribunals carried out in Mexico (Weaver, 2011), the intention in carrying out the Peoples’ International Health Tribunal was to serve as a tool for asserting justice at the grassroots level through the synthesis of multiple forms of knowledge and lived experience. Conversations with local community members had emphasized the importance of increasing awareness of local realities and experiences of community members living in close proximity to mining operations. Initial planning with community members had focused on deciding on a research deliverable that could have both local and international impact. Furthermore, it was stressed that the local community needed to feel that the benefit of such an activity was tangible and accessible.

The Peoples’ International Health Tribunal was carried out on July 14th and 15th of 2011. With 600 people in attendance daily, including “jury members” and expert witnesses throughout the Americas (e.g., Canada, Guatemala, Mexico, Chile, United States, Honduras, El Salvador) and national and international media coverage, the event on many accounts, was considered a success. Perhaps most importantly, community members from San Miguel Ixtahuacán, as well as from Carizalillos, Mexico, and Valle de Siria, Honduras, all communities in close proximity to Goldcorp operations, were all able to exchange their experiences and struggles through personal *testimonios.* These *testimonios* highlighted social, environmental, and health threats experienced by community members that often resulted in psychological, emotional, and physical health challenges. The final day of the tribunal, the jury delivered a guilty verdict to Goldcorp as they found the company guilty of health, environmental, and human rights violations throughout MesoAmerica. The verdict received local, national, and international coverage including community radio stations, national newspapers (e.g., *Prensa Libre*), continental-wide mediums (e.g., TeleSur) and international news sites (e.g., Upside Down World, *Forbes* Magazine). The verdict stated (www.healthtribunal.org/the-final-verdict/)

. . . we find Goldcorp guilty for its activities in Honduras, Guatemala and Mexico, which we find to be seriously damaging to the health and the quality of life, the quality of environment, and the right to self-determination of the affected Indigenous and campesino communities.

We also find the States where the accusations come from guilty of being complicit and irresponsible for not protecting the rights of those affected by mining.

We also find the Government of Canada guilty for supporting and promoting in various ways the irresponsible mining investments in Mesoamerica.

In this sense, The Peoples’ International Health Tribunal was both complimentary and an alternative to more conventional knowledge translation activities. It cohesively addressed community priorities of justice-seeking, local-benefit, and raising international awareness.

In the following section, I will provide some reflections on the Peoples’ International Health Tribunal. I will discuss how the planning and carrying out of this event can be conceptualized as a key decolonizing, participatory community-based narrative research act as demonstrated through mechanisms of (a) *restorying representation in relationships* and (b) *analysis as mobilization*. In this way, I seek to describe how Indigenous-informed storytelling and participatory research mechanisms, can work synergistically to build collaborative, rigorous, and dynamic insights important to better understanding the health of diverse communities. Then, I will discuss recommendations for Indigenous/community-based health scholars seeking to carry out participatory, Indigenous-informed storied research.

### Restorying Representation in Relationships

The Peoples’ International Health Tribunal served as an alternative forum to showcase voices and perspectives of community members brought to light throughout previous research activities and day-to-day life. This event was framed by principles of PAR as the research process by definition needed to transform findings into actionable-knowledge of direct benefit to local community members. Furthermore, through the planning and execution of the event, community members and myself were active in storying and restorying, reflecting decolonzing acts of framing meaning, intention, process, and the nature of stories themselves. As the research process was intended to be collaborative and participatory, planning throughout the research process, including the health tribunal, also raised issues of community ownership, representation, benefit, and reciprocity. These questions in turn, further enriched our reflexive journey toward community-based storytelling.

**Figure 2.**
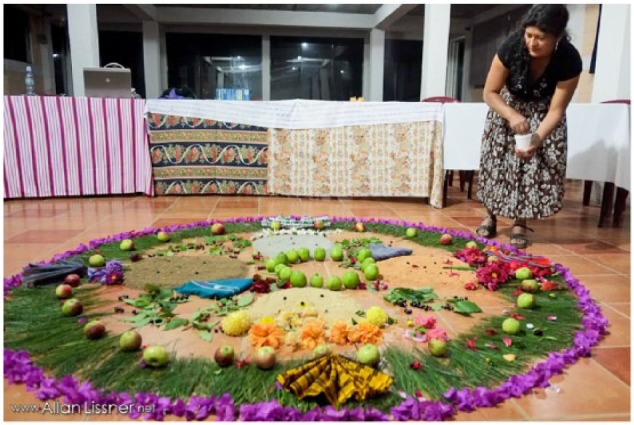
Preparing Mayan Mam altar for the Peoples’ International Health Tribunal. Source: http://allan.lissner.net

For example, in preparing the space where the tribunal would take place, a spiritual leader shared that there was a need to “decolonize” the room—pointing to straight lines of chairs assembled in the salon. As a group, we rearranged the chairs that had been in rows into a circular formation. In the middle of the space, community leaders arranged a Mayan Mam altar with candles lit in each of the four directions (see Figure 2). With this space overtly Indigenized to reflect a Mayan Mam worldview, new stories and possibilities were bound to emerge. In this way, the health tribunal space shared by the audience, affected community members, leaders, and judges was concretely conceptualized as, or, grounded in a Mayan Mam place of meaning.

During the health tribunal, community members provided testimonies in their own words of how the presence of this gold and silver mining company was affecting their health and well-being. Unlike more formal academic forms of dissemination, the researcher’s language and interpretations were not foregrounded. Directly inverse to traditional qualitative research reporting, it was the researcher’s voice, not the participants, that would serve as a resource, one of many voices, complimenting and enhancing the larger narrative that community members (not the researcher) had structured. This allowed for emotive communication in relationship to other participants. These testimonies were uniquely stirring, bound up in personal pain, political struggle, and spiritual analysis, yet always inviting and meant to be heard by fellow community members as well as a wider audience. Explaining frustration over the inaction of some leaders to speak up against the mine and the damages it had caused to the natural environment and the community, one man stated (audience responses are in italics and brackets)

**Table:** 

Nuetros! [con incredulidad]	Neutral! [in incredulity]
Yo no entiendo [esto]	I don’t understand [this]
¿Es vida, es muerte?	Is it life, is it death?
¿Es cielo o es infierno?	Is it heaven or hell?
*(Eso, asi es!)*	*(That’s right, that’s how it is!)*
Hoy nosotros de FREDEMI	Today we of FREDEMI^a^
Decimos a la empresa	We say to the company
Que nos deje en santa paz	That they leave us in holy peace
Y ellos que se vayan en paz	And that they leave in peace
. . . que vayan a sus tierras	. . . that they go back to their land
. . . que se vayan a sus paises	. . . that they go back to their countries
que dios les perdone, que dios les bendiga	that God forgive them, that God bless them
pero antes que pagen [por] los	but before that they pay [for] the
daños mortales de la creacion	mortal damages to creation
de la madre naturalesa *(aplausa)*	of mother nature *(applause)*
nosotros de FREDEMI vamos a	we of FREDEMI are going to
seguir la lucha	continue the fight
porque nuestra lucha es defender la humanidad	because our struggle is to defend humanity

a Frente de Defensa Miguelense (roughly translates to Coalition for the Defense of San Miguel Ixtahuacán).

While this transcription does not capture the full emotive nature of this man’s testimony, the audience responses speaks to the way in which his reflections are resonating at the collective level. He also expresses Mayan Mam values and social justice principles of community accountability and humankind’s responsibility and reciprocal relationship to the land and to one another (personal communication, M. Lopez, 2011, 2012, 2013). This came through in several community members’ testimonies, indicating that a Mayan Mam ontology and principles of interconnectedness and reciprocity were central to not only the story’s message (i.e., content) but also how the story itself should be understood, that is, as a collective story. This was evident in several testimonies that demonstrated a role as an assertive victim^1^ as well as a compassionate observer.

One woman, as she discussed water quality concerns and consequent health issues, increased gendered violence as well as general social conflict occasioned by the presence of mining operations declared

**Table:** 

No solamente entri mi comunidad	Not only in my community
[i.e., aldea]	[i.e., village]
Pero en nuestras comunidades	But in our communities
de San Miguel [Ixtahuacan]	of San Miguel [Ixtahuacan]
. . . En nuestras familias	. . . In our families

In this sense, community members spoke of injustices and harms simultaneously as community advocates and aggrieved individuals. It was often important to stress that this was a community-level problem. As I have mentioned in a previous writing, the importance of the collective nature of experiences demands that we consider the risk of collapsing this story into an individual narrative framework as it can silence much of the intention behind the sharing of the story (Caxaj & Berman, 2014). Notably, testimonies from Mexico and Honduras similarly reflected this collective-level analysis, asserting moral agency in identifying injustices faced by their community and continuing the *restorying* of the health experiences of mining-affected communities. A Honduran woman pointing to the physical health problems among children arising from the presence of mine contaminants noted, “if this is what is called development, that I do not understand.”

This tone of radical relationality was largely determined by the Indigenous framework in which the tribunal was situated. For instance, carrying out spiritual protocols, the articulation of issues in both Mayan Mam and the Spanish language, and the sharing of traditional music (i.e., the marimba) and lyrics asserting a profound relationship to Mother Earth all framed the public discussion. Both to the larger public and the local participants, it communicated a sense of Indigenous ownership, the authority of Mayan Mam knowing, and a sense of humility in regard to Mother Earth. This framing invited others to join and compliment this discourse as allies (e.g., identifying complicity of nation-states) and participants (e.g., joining in spiritual rituals to promote fairness in deliberation) toward a more holistic view of the issues at hand.

The centrality of honoring relationships and seeking connection was also reflected in the building of networks across affected communities/organizations enabled by the tribunal. This was often a process of knowledge exchange and collective analysis, motivating further action, solidarity, and the solidifying of alliances. This interrelated movement toward action will be discussed in further detail below.

### Analysis as Mobilization

Guided by a decolonizing narrative approach, I sought to champion local Indigenous knowledge and the co-construction of findings throughout the research process. In keeping with the PAR approach, the research was intended to develop meaningful partnerships toward shared findings and community deliverables. Through focus group and one-on-one interviews, I worked with community members to engage them in the analysis by reporting back preliminary impressions (potential themes, overviews of previous interviews) before initiating a new conversation. During this time, participants would provide feedback, identify key ideas that required refining, changing, or addition, and emphasize themes or ideas that resonated with them. This would spur new conversations, allowing us to discuss key issues in greater depth, elevating the richness and complexity of mutual understanding. To illustrate, before initiating a second visit, I would report back a summary, somewhat of a running list of issues that a group had discussed, such as familial violence, bullying, poverty, and gendered exclusion. Participants would add to, emphasize (e.g., “that’s important,” “this is the problem that we are seeing in our community”) or revise (e.g., “it’s not just that we are divided, it’s that we don’t have a friendship or love for one another,” “and then there’s the contamination, I worry about the future . . . ”). Through these continued conversations, participants would reach a consensus as to the key underlying issues driving these concerns. For instance, how the presence of local mining operations, enabled by state discrimination, had resulted in social divisiveness and mistrust.

Yet as we delved deeper into collaborative analyses activities, engaging in reciprocal efforts for meaning, conversations would organically shift toward purpose, intention, and action. Discussing concerns for the natural environment, increased alcoholism, economic disparity, or violence with the arrival of mining operations for example, often concluded with reflective questions (e.g., What can we do about it? How can we make them see?) or evaluative statements toward action (e.g., it will be the children who will suffer the most, that’s why we have to stay in the struggle). In a sense, these reflections were what enabled a more complex analysis, as co-constructed knowledge and findings were understood as the basis for political action and resistance, posing a collective question to all involved—how can we use our knowledge in the service of our/the community?

**Figure 3.**
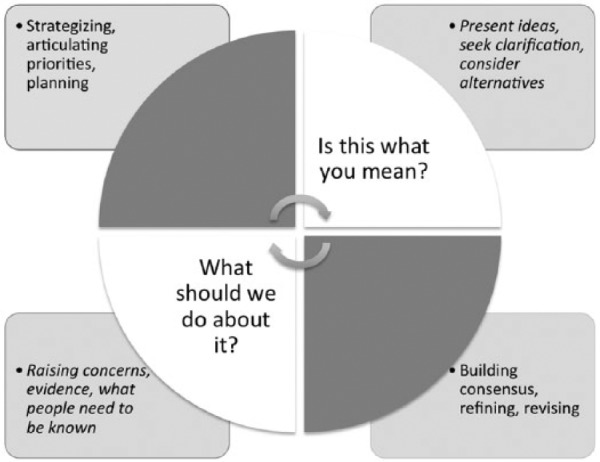
Analysis and mobilization through iterative reflexivity.

As illustrated in Figure 3, this cycle of meaning-making, actionable-knowledge is a form of deliberative dialogue that can be understood as a mechanism of *iterative reflexivity*. It is important to note that this form of collaborative analysis demanded higher reflection and accountability of both community participants and myself, the academic. Furthermore, it was clear that this relational commitment was shaped by a local Mayan Mam vision of shared accountability and ancestral teachings. For instance, one elder shared with me his experience in grade school of being hit whenever he spoke in Mam to his peers. He taught me about the practice of visits and hospitality, and generosity, even in moments of scarcity. Through his friendship and that of others, I was invited to explore my relationship to the community as a Quiche/Kachiquel/Mestiza woman displaced during the 1980s and to reflect on our shared history of state uprooting. That is, the exchanging of stories propelled a collective understanding of one another and our obligation and responsibility to contribute to our decolonization and continued legacy of our ancestors. These exchanges of history, moments of connection, and consensus-building provided the initial momentum to continue to consult with the wider community and slowly develop an agreed on framework to move forward with the planning of what would become the Peoples’ International Health Tribunal.

Analysis was not restricted to private research spaces. In fact, both community planning and the realization of the health tribunal served to validate emerging findings and add emphasis to key messages. Of equal importance, community members’ testimonies and ultimately, the judges’ verdict, provided a medium to express support and solidarity among affected communities by providing important starting points to build coalitions and connections. As testimonies were shared from communities throughout Latin America and Guatemala, these two mechanisms (i.e., validation/emphasis, support/solidarity) worked in synchrony to promote a larger nation-wide or continental analysis of the issue of gold mining’s impact on health. This in turn, motivated a more purposeful, contextually grounded analysis bound to personal relationships, Mother Earth and social justice. Sometimes, testimonies provided an opportunity for community members to express their commitment to their community and honor their fellow community members who had been active in speaking out about injustices. One woman commented,

**Table:** 

Nosotros seguimos al pie	We continue on our feet
. . . a pesar de las calumnias	. . . despite the lies
. . . a pesar de los maltratos	. . . despite the abuses . . .
pero sabemos que nuestra lucha es	but we know that our struggle is
Transparente	Transparent
porque solo estamos defendiendo	because we are only defending
nuestro territorio . . .	our territory . . .
because . . . nuestra lucha	because . . . our struggle
es para defender nuestra vida	is to defend our lives
pero no solo eso, pero la vida de todos	but not just that, but [also] the life of everyone

A man from the neighboring municipality of Sipakapa stated

**Table:** 

From Sipakapa, animo!	From Sipakapa, courage!
El pueblo unido, jamas sera vencido	The people united will never be defeated
Estamos en lucha	We are in the struggle
Mientras estamos en el frente	as long as we are at the front

In this sense, testimonies provided a unique formal medium for community members to support one another, bear witness to each other’s suffering, and inspire one another to persevere. This was felt by both individuals providing testimonies as well as by the general audience as many community members provided feedback that their spirits had been lifted, or, they had been re-energized in participating in the health tribunal.

Both participation through testimonies as well as in interviews with journalists functioned as storied public analyses, engaging a wider audience to consider the health threats caused by precious metal mining. Voicing these concerns often led speakers to articulate a profound impetus for social justice to catalyze international actors to challenge injustices in the extractive industry. In discussing the colonial roots and systemic injustices shaping the experiences of mining-affected communities in Guatemala, one woman appealed to individuals and organizations to recognize the urgent nature of the health threats posed by large-scale mining. She stated

**Table:** 

Ojala que ellos escuchen	Hopefully they will listen
Entiendan lo que – los testimonios	That they understand—the testimonies
que estamos dando aqui en SMI [San Miguel Ixtahuacán]	that we are giving here in SMI [San Miguel Ixtahuacán]
y tambien que ellos se levanten porque	and also that they rise up because
no solo aqui existe el oro – si la	the gold does not just exist here—if the
empresa va explotar al nivel de	company is going to exploit at the level of
Guatemala, donde iremos?	Guatemala, where will we go?
Nos quieren quitar nuestras	They want to take our
tierras, nos quieren quitar nuestras vidas	lands, they want to take our lives
con, nos quieren matar	with—they want to kill us
con las contaminaciones	with the contaminations
. . .entonces, esa es la funccion	so then, this is the purpose
de la empresa	of the company
acabarnos como pueblos indigenas	finish us as Indigenous peoples
y quedar con nuestras tierras	and keep possession of our lands
quedar con todo nuestros	stay with all our
recursos naturales, pues yo diria que	natural resources, so I would say that
todos se levanten y que	everyone rise up and that
nadie se quede atraz	no one stay behind
para unirons en la lucha para sacar	to unite in the struggle to remove
todas las empresas mineras	all mining companies
que existen en nuestro pais	that exist in our country

In closing, the Peoples’ International Health Tribunal delivered a guilty verdict, making the following recommendations:

Of the States (national, departmental, state, and municipal governments)

- Compliance with existing national legislation and international agreements, in particular, those that guarantee the right to free, prior, and informed consent.
- Creation of new regulations to protect and guarantee the rights of communities who are confronting mining and all activities that affect their well-being.
- Emphatically ensure the respect for and enjoyment of the rights of indigenous peoples, recognizing their own traditions, cultures, and decision making.
- Adopting measures similar to the restrictions on open-pit metallic mining decreed by the authorities of other countries.

Of Goldcorp, we demand

- Reparations of the damages to the health of the population, the damages to the environment, and in general damages to the affected indigenous and peasant communities.
- Compensation for past, present, and future damages to the communities, taking in consideration that contamination is ongoing and can continue still for hundreds of years.
- Suspension of all mining operations in Mesoamerica and guarantees that it will not repeat the experiences described in the accusations herein.

Thus, through the verdict itself, the health tribunal continued to promote a collective analysis, seeking to mobilize change and social justice. Ultimately, these declarations were spurred by the wisdom, perseverance, and collective spirit of the Mayan Mam peoples of San Miguel Ixtahuacán.

## Discussion and Reflection

As a displaced Mestiza woman of Quiche and Kachiquel descent and (assumed) European ancestry, with a Western educational background and Canadian citizenship, I came with unique privileges, oppressions, and contradictions in seeking to carry out research with a mining-affected Mayan Mam Indigenous community. I have discussed some of the challenges and opportunities of doing collaborative research, given my particular identity in previous publications (Caxaj et. al, 2012; Caxaj & Berman, 2014) It is important to note however, that my various social positions bridged, shaped, and at times, limited my ability to work in full partnership with community members. Absolon and Willett (2005) note that identifying and discussing social location is a methodology in and of itself, central to decolonizing research processes. Needless to say, sharing my experiences of privilege and oppression, for example, sharing my familial experiences as a refugee as a result of Guatemala’s violent history and my ease traveling to and from Guatemala with a Canadian passport, were important to working toward transparency and trust throughout the research process (see Caxaj & Berman, 2014 for further detail). Given a 36-year state-led war that killed or led to disappearance of 20,000 and displaced 1.5 million of which 83% of victims were Indigenous, all under the rhetoric of “national unity” (Guatemalan Commission for Historical Clarification (CEH), 1999; Oficina de Derehos Humanos del Arzobispado de Guatemala, 1998), voicing awareness of difference and the lived realities of such state violence is a powerful act. At best, perhaps it can enable a collective understanding of how diverse Guatemalans and others can work in solidarity, and at a minimum, build spaces conducive for increased accountability. I strived to contribute to these processes by recognizing the limitations of conventional academic constructs. And instead, worked to develop a collaborative research process with the Mayan Mam community of San Miguel Ixtahuacán that would build new understandings of both the research process and final outcomes. We carried out the Peoples’ International Health Tribunal with an overt intent to address power differentials while honoring unique local knowledges and experiences toward a shared vision of empowerment and change.

According to Baum, MacDougall, and Smith (2006), a PAR framework is distinct from other research approaches in that it (a) is focused on generating research to promote action or change, (b) is centered on decreasing power differentials and power-sharing, and (c) seeks to directly involve community participants in the research. A PAR framework enabled necessary conversations and spaces by acknowledging differences in power and priorities between community members and the researcher, demanding a more flexible and democratic realization of research activities. In being community-informed and collaborative, research processes ensured more diverse, rigorous, and accountable findings and understandings. Key to the success of these strategies was the building of relationships, the honoring of difference and epistemological pluralism, and a commitment to demonstrating accountability and reciprocity. As noted by Indigenous scholars, Western research is seeped in a monolithic understanding of knowledge that assumes individual ownership of knowledge enabling exploitative practices that can co-opt and distort Indigenous ways of knowing (Kovach, 2009; Smith, 1999). Thus, addressing issues of power, privilege, and representation are key to building co-constructed narratives. The tribunal sought to interrupt Western readings of community health concerns by asserting the authority of Indigenous voices, local experiences, and community justice by mobilizing public analyses, key witness accounts, and denouncements. This involved active steps toward power-sharing and making space for community participation, but of equal importance, there was a need to overtly acknowledge the distinct knowledge base of participating communities. Storied processes throughout data collection, analyses, and the sharing of *testimonios* enabled rich dialogue of unique local knowledge(s) and ontologies and the historical forces that shape them.

*Testimonios* are powerful forms of storytelling central to truth-telling, justice-seeking, and bearing witness that have a long history throughout Latin America. Given the history of genocide and conflict targeting Indigenous populations in Guatemala, and, the systemic denial and neglect surrounding this violent legacy, community efforts to voice every-day experiences are often, inevitably political. Awareness of this strong oral history imbedded in historical memory of loss and colonial violence, indicate the importance of the *traditional*, spiritual, and/or the ritual in justice-seeking practices among some Indigenous populations throughout Guatemala. As testimonios are informed by specific local contexts and histories, there is a need to honor the epistemic roots of this storied knowledge instead of simply conflating this practice within a Critical paradigm. As noted by Bowers (2003), a danger with a Critical approach is that aims to “rename,” “transform” or “change,” if unchecked, can align more with a colonial legacy that normalizes dispossession, assimilation, or appropriation for the sake of ”progress” or “enlightenment.” Scholars wishing to be allies to Indigenous communities must define and redefine what is meant by “change” or aims of empowerment in a way that is central to local Indigenous ontologies and the community’s priorities.

Many community testimonies were expressed as collective or shared experiences, intertwined in relationship with one another, the wider community, and the natural environment. As I have previously mentioned, in honoring and making room for the expression of Indigenous ways of knowing, it is necessary to recognize that many readings of Western scholarship are at odds with local Indigenous knowledge systems. When these incommensurable analyses emerge, it is the responsibility of scholars and other allies alike, to demonstrate a readiness to be flexible and open to different possibilities for carrying out a project. This is not to say that differences will ever be “resolved” but recognizing and addressing these ontological tensions can produce stronger and more ethically profound research scholarship. In this particular research process, we were able to identify the limitations of more conventional knowledge translation activities (e.g., written texts, individual analyses), and engage instead, in a community-based event that honored local knowledge and oral accounts of collective experiences. Consistent with the writing of other Indigenous scholars, this process highlighted principles of collectivity, hope, spirituality, interconnectedness, and tradition as key to Indigenous ways of knowing (Castellano, 2008; Grande, 2008).

The Peoples’ International Health Tribunal serves as a strong exemplar of how research projects can be profoundly strengthened by the incorporation of local Indigenous knowledge throughout the research process. Yet this incorporation is only possible when the scholar is actively aware of how this knowledge is embodied, and as such, must occur through a process of relationship and transparent knowledge exchange. It cannot simply be a superficial step (add a dash of Indigenous and stir) as this will result in tokenizing, little community benefit, and exploitative research activities. In other words, Indigenous-based approaches are not merely a “gesture,” but instead, a reframing and reorienting of the research itself toward Indigenous control, ownership, and self-definition (Poulani Louis, 2007). In this sense, PAR principles provide a strong framework to build decolonizing storied narratives through meaningful partnership with communities. This process, can illuminate ways in which methodological and ethical research concerns are often arbitrary distinctions because engaging in activities with a community will simultaneously build trust, respect, and rich understandings. In this sense, an ethic of storytelling can help fuel a more robust participatory research framework further accountable to community priorities.

It may be important for critical and/or decolonizing researchers to map out how their analysis is action-informed and vice versa (how their action is analysis-informed) as a way to demonstrate how activities and events that are viewed as “unconventional” and outside of the research project, are in fact, central to the research process. This may also help provide more critically oriented or decolonizing guidelines for scholars wishing to develop integrated knowledge translation processes while remaining true to their paradigmatic orientations. Furthermore, by, identifying the intrinsic relationship between action and analysis, as illustrated in the conceptualization of *iterative reflexivity*, we may more concretely honor the voices of the communities we are working with, recognizing that the meanings and realities they express are of inherent value with or without an academic framing. Otherwise, researchers wishing to carry out emancipatory research may unwittingly reinforce hierarchies of knowledge and marginalization of voices by conforming strictly to conventional academic constructs of scholarship. Myers (1994) further warns that critical scholars solely focused on an ideology versus the storied meaning conveyed by an Indigenous storyteller may contribute to “double erasure of agency,” first brought about by colonial mechanisms. The point here is not to argue that there can be such a thing as a “pure voice,” outside of the research process but simply to argue for the need to remain committed to building reciprocity and partnership into meaning. Key to this commitment is a practiced awareness of the agency of communities in expressing and constructing multiple truths. According to Soyini Madison (2008), the researcher can contribute to co-constructions of narratives without silencing, co-opting, or distorting community voices. While this is a delicate balance, she notes that a researcher’s analysis can serve (a) “ . . . as a magnifying lens”; (b) “ . . . to clarify and honor the significance of the telling . . .”; and (c) “ . . . to unlock the multiple truths . . . below the surface” (Soyini Madison, 2008, p. 294). Yet ultimately, “the subaltern does speak, always, and we must listen with more radical intent” (Soyini Madison, 2008, p. 295). Through carrying out the Peoples’ International Health Tribunal, we worked to build spaces to honor the significance of community members’ stories and to build connections and networks of meaning among communities affected by large-scale mining.

In conclusion, there is great potential in using both PAR and narrative/storytelling strategies toward a decolonizing storied participatory approach. Co-constructing meaning is both a process of content and form. When working with Indigenous communities, unique ontologies will shape how stories are understood, framed, and told. In this particular case, principles of interconnectedness and collectivism were central to meaning-making. Furthermore, stories were important in building relationships, strengthening communities, and broadening networks of support, *restorying stories in relationships.* Principles of PAR helped enable formal spaces to ensure that these types of stories could be told and to continue conversations beyond strictly academic spaces. Ongoing conversations and analyses through stories and knowledge exchange also enabled more comprehensive understandings closely linked to building higher accountability to community needs and priorities. This was achieved through cyclical acts of *iterative reflexivity* that propelled analysis as a mechanism for mobilization. In this sense, a storied approach was instrumental to building an authentic application of PAR principles into the project toward higher methodological and ethical accountability. More discussion is required to better guide novice and seasoned scholars alike to build from these two approaches in collaboration with community partners.

## Notes

1. See Caxaj & Berman, 2014 for further discussion of challenging the binary of agency and victimhood
